# Cellular Signaling Pathway Alterations and Potential Targeted Therapies for Medullary Thyroid Carcinoma

**DOI:** 10.1155/2013/803171

**Published:** 2013-02-21

**Authors:** Serena Giunti, Alessandro Antonelli, Andrea Amorosi, Libero Santarpia

**Affiliations:** ^1^Department of Pathology, Centro Oncologico Fiorentino, Sesto Fiorentino, 50019 Firenze, Italy; ^2^Department of Internal Medicine, University of Pisa School of Medicine, 56100 Pisa, Italy; ^3^Translational Research Unit, Department of Oncology, Istituto Toscano Tumori, 59100 Prato, Italy

## Abstract

Parafollicular C-cell-derived medullary thyroid cancer (MTC) comprises 3% to 4% of all thyroid cancers. While cytotoxic treatments have been shown to have limited efficacy, targeted molecular therapies that inhibit rearranged during transfection (RET) and other tyrosine kinase receptors that are mainly involved in angiogenesis have shown great promise in the treatment of metastatic or locally advanced MTC. Multi-tyrosine kinase inhibitors such as vandetanib, which is already approved for the treatment of progressive MTC, and cabozantinib have shown distinct advantages with regard to rates of disease response and control. However, these types of tyrosine kinase inhibitor compounds are able to concurrently block several types of targets, which limits the understanding of RET as a specific target. Moreover, important resistances to tyrosine kinase inhibitors can occur, which limit the long-term efficacy of these treatments. Deregulated cellular signaling pathways and genetic alterations in MTC, particularly the activation of the RAS/mammalian target of rapamycin (mTOR) cascades and RET crosstalk signaling, are now emerging as novel and potentially promising therapeutic treatments for aggressive MTC.

## 1. Introduction

Medullary thyroid carcinoma (MTC) is a rare neuroendocrine cancer that originates from thyroid parafollicular calcitonin-(CT-) producing cells. MTC accounts for approximately 4% of all thyroid malignancies; approximately 75% of these cases occur in the sporadic form, and 25% occur in the hereditary form [1–3]. MTC usually has a favorable prognosis, with a 10-year survival rate of 70%–80%, if it is diagnosed and treated at an early stage when the tumor is confined to the thyroid [4]. Unfortunately, most cases of MTC present at diagnosis with metastases to the local and regional lymph nodes and to distant organs, especially the lungs, liver, and bones [5]. Patients with metastatic MTC have a 10-year overall survival rate of 40%, and metastasis is the main cause of death in patients with MTC [4, 6]. Locally advanced and distant metastatic diseases are incurable, as surgical resection and conventional radio- and cytotoxic chemotherapies are not effective against metastatic MTC [7, 8]. Clinical trials of various combinations of chemotherapeutic drugs have yielded unsatisfactory results [9, 10]. However, research over the last years has led to a good understanding of the genetic defects and altered molecular pathways that are associated with the development of MTC. Thus, multiple promising therapeutic agents that target these genetic alterations have been developed to treat progressive and advanced MTC. Activating mutations of the tyrosine kinase receptor (TKR) rearranged during transfection (*RET*) are believed to be the primary oncogenic event in a majority of MTC cases. This discovery has led to the development and introduction of targeted therapies, such as tyrosine kinase inhibitors (TKIs) that target RET. Several TKIs directed toward RET kinase have been tested *in vitro*, preclinical, and clinical studies with promising results. Unfortunately, these agents are not likely to be curative, as the longest duration of response observed was approximately 4 years, and the maintenance of agent-dependent effects may require continuous therapies [7], which are not without important side effects. The main reasons for the failure of these agents to cure MTC are the development of resistance to TKIs that target the RET and other cell receptors and the activities of other signal transduction pathways that are involved in MTC tumorigenesis and progression but not directly targeted by TKIs. In recent years, the discovery of mechanisms of resistance to TKIs and of several other molecular events that contribute to MTC transformation and metastasis suggested that combinatory therapy may result in a more significant tumor growth inhibition. This has led to the development of novel compounds that have been used in several clinical trials, including TKIs that can target multiple TKRs simultaneously in addition to RET and agents that can target other altered signaling pathways. Other studies have demonstrated the potential for immunotherapy in combination with agents that target signal transduction pathways that are important for MTC growth [5]. Because the aim of these targeted therapies is to extend lifespan and increase the quality of life, it is very important to limit the toxicities of therapeutic agents, either alone or in combination. The possibility of testing these novel drugs* in vitro* (in primary thyroid cancer cells) and *in vivo* may help to improve the personalization of treatments [11].

## 2. Key Cellular Signaling Pathways and Alterations in MTC

### 2.1. RET Pathway

The role of the *RET* oncogene in the tumorigenesis of MTC has been characterized extensively [12]. The *RET* gene encodes a transmembrane tyrosine kinase that binds to glial cell line-derived neurotrophic factor (GDNF) family ligands [13]. RET signaling leads to the activation of the RAS/mitogen-activated protein kinase (MAPK) and the phosphatidylinositol 3′ kinase (PI3K)/Akt pathways and has key roles in cell growth, differentiation, and survival. Activating point mutations of the TKR *RET* have been reported in nearly all hereditary cases of MTC; some of these mutations are included in the MEN2A, familial MTC, or MEN2B syndromes in which there is a genotypic/phenotypic correlation between the type of *RET *mutation and clinical features. *RET* mutations are also found in 30%–50% of sporadic MTCs. Germline mutations in the *RET* proto-oncogene are responsible for hereditary MTC, while somatic *RET* mutations are responsible for sporadic MTC [14]. These data provide a strong rationale for targeting RET in selective cancer therapy. However, this paper will mainly focus on additional cellular signaling pathways other than RET responsible of MTC tumorigenesis and progression and potential targeted approaches for the treatment of advanced or metastatic MTC.

### 2.2. Additional Signaling Pathways That Accelerate MTC Progression

Although activating mutations of the TKR *RET* are believed to be the primary oncogenic event in the development of a majority of MTC cases, it is clear that RET cooperates with other signal transduction pathways to promote MTC tumorigenesis.

#### 2.2.1. Tyrosine Kinase Receptors other than RET Are Implicated in MTC Tumorigenesis

In addition to RET, other kinase receptors may play a role in the development and progression of MTCs [15]. 

Similar to the RET receptor, the epidermal growth factor receptor (EGFR) is a TKR that is associated with the regulation of cell growth, proliferation, and apoptosis. Dimerization of the receptor following ligand binding results in transphosphorylation and the subsequent activation of several downstream signal pathways. EGFR has been shown to be frequently overexpressed in various types of thyroid carcinomas, including MTC, and to play a role in cancer development and progression [16]. In contrast, a recent report analyzing different MTC on tissue microarrays has demonstrated that only 20% of cases revealed moderate to strong reactivity for EGFR, whereas the majority of the cases revealed weak and very focal positivity [17]. A different study has shown that *EGFR*-activating mutations are rare in MTC. With respect to the numbers of *EGFR* gene copies in MTCs, the researchers did not detect amplifications but did find polysomes in 15% of the examined tumors [18]. Additionally, EGFR was activated in a subset of MTCs, which suggests that this subset of patients might benefit from drugs that target also EGFR. Recent findings have shown that the ligand-induced activation of EGFR can stimulate RET activation beyond signaling and growth stimulation [19]. Several EGFR inhibitors have been shown to markedly inhibit the growth of the MTC TT and MZ-CRC-1 cell lines. Because RET activation seems to be influenced by EGFR, a recent study investigated whether EGFR activation could be related to specific *RET* mutations in MTCs. The researchers found that tumors with the most aggressive *RET* mutations (in codons 883/918) exhibited reduced EGFR expression compared to other *RET* mutations. It could be speculated that the most aggressive *RET* mutations are less dependent on EGFR activation [18]. In fact, in the work by Croyle et al. [19], in which cell lines with *RET* mutations in codon 634 and codon 918 were compared, the effect of EGFR inhibition on the codon 918 mutated cell line appeared to be reduced, in agreement with the previous data. Because the activation status of EGFR seems to be related to RET activation, EGFR activation has been examined in *RET*-negative tumors [18]. However, no differences have been found in EGFR activation between RET-positive and -negative tumors, which likely indicates that other molecular mechanisms lead to RET activation, such as increased *RET* gene copy numbers, altered promoter activity, or increased transcription in the *RET* mutation-negative tumors. These data suggest that EGFR status determination in MTCs might be important but certainly deserves further investigations.

The vascular endothelial growth factor receptor (VEGFR) pathway is also important in the pathogenesis of MTC. There are three transmembrane receptors that mediate the angiogenic and lymphogenic effects of VEGF: VEGFR-1, VEGFR-2, and VEGFR-3. VEGFR-2 is thought to be implicated primarily in tumor growth and metastasis. Overexpression of VEGF and VEGFR-2 has been found in MTC compared to normal thyroid tissue [20]. The VEGF proteins (VEGF-A, B, C, and D), which are secreted by tumor cells, act as ligands for the VEGFR-2 receptors on endothelial cells and promote a signaling cascade through different pathways, such as PLC-*γ*-PKC-Raf-MEK-MAPK and PI3K-Akt, that stimulate cellular proliferation, migration, and survival and induce neoangiogenesis [21]. Angiogenesis is an essential alteration in cell physiology that predisposes the development of malignancy and is fundamental in tumor growth and metastasis [22]. Similarly to EGFR, the overexpression of VEGFR-2 in MTC has been shown to correlate with metastasis [18]. 

Several multitargeting tyrosine kinase inhibitors that block VEGFR have shown promising clinical antitumor activity; unfortunately, in most thyroid carcinomas and other solid tumors, the antiangiogenic effects are often only transitory and really often may have late paradoxically protumorigenic effects. Additionally, it seems that a modest significant association has been observed between VEGFR-2 expression and *RET *mutation status in primary tumors [18]. 

The *MET* proto-oncogene codes for the TK receptor for the hepatocyte growth factor (HGF). The HGF-MET interaction activates signaling pathways that mediate cell adhesion and motility. MET hyperactivation reportedly correlates with the metastatic abilities of tumor cells [23]. MET and HGF coexpression has been observed in a subset of MTC tumors and is associated with multifocality in MTC [24], which makes this interaction a potentially important target. In one report, mutations in the *MET* RTK were detected in MTC [25]. Importantly, RET can induce the overexpression of c-MET in this type of thyroid tumor [26]. 

The fibroblast growth factor receptor 4 (FGFR4) has also been reported to be overexpressed in MTC cell lines. Inhibition of FGFR phosphorylation with the small molecule FGFR inhibitor PD173074 resulted in an arrest of cell proliferation and tumor growth [27]. Moreover, the dual inhibition of RET and FGFR combined with tyrosine kinase inhibitors resulted in greater suppressions of cell proliferation *in vitro* and tumor control *in vivo* than that which was achieved with either agent alone. These data highlight RET and FGFR4 as therapeutic targets and suggest a potential role for the use of combined tyrosine kinase inhibitors in the management of inoperable medullary thyroid cancers [28].

Finally, the platelet-derived growth factor receptor (PDGFR) also seems to play a role in differentiated thyroid cancer [29], although its role and function have not been fully investigated in MTC.

#### 2.2.2. Other Signaling Pathways That Contribute to MTC Tumorigenesis

Several other signal transduction pathways have been implicated as contributors to MTC tumor growth, as illustrated in two recent studies [5, 30]. These pathways include RET interactions with pRB, p53, p18, and p27 as well as the phosphatidylinositol 3-kinase/AKT/mTOR and Ras/Raf/MEK/ERK pathways.

*RET Interactions with Tumor Suppressor Genes*

*(a) pRB and p53.* The tumor suppressor genes *RB1* (retinoblastoma; pRB protein) and *TP53* (p53 protein) are frequently mutated in human cancers, and it appears that, in cancer, both pathways must be inactivated to overcome senescence or apoptosis. There is extensive genetic evidence that the pRB and p53 pathways are involved in MTC in rodents. For example, *RB1*-deficient mice developed MTC [31]. Further, the loss of *TP53* further increased MTC formation in *RB1*-deficient mice [32, 33]. It has been shown that in mice, RET cooperates with the inactivation of pRB/p53 to cause experimental MTC. pRB^+/−^/p53^+/−^ mutant mice have been shown to acquire *RET* mutations that are analogous to activating germline mutations that are observed in human MEN2A and familial medullary thyroid carcinoma (FMTC). This suggests that murine MTC requires mutational dysregulations within both the *RET* and nuclear tumor suppressor gene pathways [34]. However, mouse models may not mimic human disease, and a systematic analysis of the genes in the *RB1* and *TP53* pathways in human samples will help to clarify their roles in human MTC formation. This information may be important for the development of novel targeted therapeutic approaches for MTC. 

*(b) p18 and p27.* Thyroid tumors show low expression of the cyclin-dependent kinase inhibitor (CDKI) p27 (Kip1), and recent evidence demonstrates that p27 is downregulated by the active RET mutant, RET/PTC1, which is found in papillary thyroid carcinomas. These data implicate decreased p27 activity as an important event during thyroid tumorigenesis. However, p27^−/−^ mice develop MEN-like tumors only in combination with the loss of p18 (Ink4c), another CDKI. This suggests that p18 and p27 are functional collaborators in the suppression of tumorigenesis, that the loss of both is critical to the development of MEN tumors, and that both p18 and p27 are regulated by RET [35]. 

*PI3K-AKT-mTOR Pathway.* The PI3K-AKT-mammalian target of the rapamycin (mTOR) cascade is important in tumorigenesis due to its ability to promote cell growth, proliferation, and survival. Several examples provide evidence to support a role for the activation of the PI3K/AKT/mTOR signaling cascade in medullary thyroid cancer [36–38]. 

Several mechanisms have been shown to be involved in the activation of PI3K signaling in medullary thyroid cancer.

A mutation of MEN2A (RET-MEN2A) has been shown to activate PI3K and its downstream effector, the serine/threonine kinase AKT/protein kinase B [35]. Previous studies have demonstrated that a mutation of Tyr-1062, which is the intracellular docking site for Shc and Enigma on RET, abolishes the RET-MEN2A transforming activity [39]. These studies further revealed that the mutation of Tyr-1062 abrogates the binding of the p85 regulatory subunit of PI3K to RET-MEN2A and subsequent stimulation of the PI3K/AKT pathway. Furthermore, retroviral transduction of rat fibroblasts with a dominant-interfering form of PI3K was shown to suppress RET-MEN2A-dependent transformation, whereas the overexpression of AKT enhanced RET-MEN2A oncogenic potential. In summary, these data are consistent with the notion that RET-mediated cell-transforming effects are critically dependent on the activation of the PI3K/AKT/mTOR pathway [36].

In cell lines, the PI3K-AKT/mTOR pathway has been shown to be important in the pathogenesis of MEN2B [40]. RET-MEN2B (*RET* M918T) is more effectively autophosphorylated at RET Y1062 than is RET-MEN2A, which subsequently leads to increased constitutive activation of the Ras/mitogen-activated protein kinase (MAPK) and PI3K/AKT/mTOR signaling cascades [41].

Furthermore, previous data report other possible mechanisms of PI3K activation in thyroid carcinomas, such as the overexpression of RAI (ShcC/N-Shc), which is a substrate of RET oncoproteins [5, 42].

Finally, the loss of expression of the phosphatase and tensin homologue (*PTEN*) gene, a tumor suppressor gene, may have a role in the activation of PI3K/AKT in thyroid tumors. The absence of functional PTEN protein expression has been observed in various cancer cells and has led to the constitutive activation of downstream components of the PI3K pathway, including the Akt and mTOR kinases. Preclinical models showed that inactivation of these kinases is able to reverse the effects of PTEN loss [43]. These data raise the possibility that drugs that target either these kinases or PI3K itself might have significant therapeutic activity against PTEN-null cancers.

Mutations in major nodes of this signaling cascade have been observed in human cancers; these mutations include gain-of-function mutations and amplifications of the genes encoding PI3K and AKT [44, 45]. No systematic genetic analysis of PI3K pathway components has been reported in MTC. However, a recent study on a series of 49 MTCs has shown that the PI3K genes were not mutated, and that the activation of the PI3K pathway was significantly associated with the status of *RET *mutations [46]. In fact, using protein expression analysis, the same authors confirmed that the AKT/mTOR pathway was highly activated in MTC, especially in cases with germline *RET* mutations. Interestingly, this association was not dependent on the type of mutation (in either the codons of the juxta-membrane or of the tyrosine kinase portion of the receptors) but was dependent on the hereditary nature of the mutation. In contrast, medullary carcinomas with sporadic *RET* mutations or with wild-type RET were observed with heterogeneous expression of AKT/mTOR pathway molecules, which suggests the need for further elucidation of alternative activation mechanisms [46]. In a recent study, the PI3K/AKT/mTOR pathway was shown to be activated in MTC, particularly in the metastatic lymph nodes, and this pathway was shown to sustain malignant features of different MTC cell models [47]. Moreover, it has been shown that the selective inhibition of the mTOR pathway in a germline-RET-mutated MTC cell line can effectively decrease cell viability and block the phosphorylated status of mTOR signaling molecules, which confirms previously published *in vitro* data [48]. Another study used metformin, which is an antidiabetic agent that decreases the proliferation of cancer cells through the 5′-AMP-activated protein kinase-dependent inhibition of mTOR, in MTC cell lines to show that the growth-inhibitory effects on the cells were associated with the downregulation of both the mTOR/6SK and pERK signaling pathways [49]. Altogether, these results strongly suggest that mTOR might be a very efficacious target in patients with advanced or metastatic MTC.

Noteworthy, mTOR inhibitors are currently being used in clinical trials for the treatment of medullary thyroid carcinoma. 

*Ras-Raf-MEK-ERK Pathway.* Unlike hereditary MTC, in which *RET* mutations are the critical events, in sporadic MTC, the genetic or molecular biomarkers have not been fully established. *Ras* is a frequently mutated oncogene in a broad spectrum of human tumors, including thyroid carcinoma, mainly in the follicular forms [50]. In this context, investigators aimed to determine whether mutations in the *Ras* oncogene could play a possible role in the carcinogenesis of sporadic MTC. The initial study analyzed 15 sporadic MTCs for mutations in known hot spots (codons 12, 13, and 61) of the *H- *and *K-Ras* oncogenes by a direct sequencing technique, although no *Ras* mutations were detected in any of the examined tumors [50]. A different study on 49 MTC confirmed that *Ras* mutations are a rare event in this type of tumor, regardless of *RET *mutational status [46]. By contrast, different studies have demonstrated the presence of *Ras* mutations in MTC. A mutational screening for *H-*,* K-*, *N-RAS*, and *BRAF* in 13 sporadic and inherited MTCs revealed one sporadic RET-negative MTC (stage III) with a mutation in the *H-Ras* codon 13 (G13R) [51]. Another study analyzed 188 hereditary and sporadic MTCs for *Ras *mutational status, revealing a low prevalence of mutations, which were confined only to RET-negative sporadic MTC [52]. Furthermore, recent studies have reported a quite high prevalence of *Ras *mutations in sporadic MTC, particularly in RET-negative MTC; 68% (17 of 25) of the RET-negative MTCs had mutations of *Ras *compared to only 2.5% (1/40) of the RET-positive MTCs [53]. These findings were confirmed by a different study that analyzed 17 sporadic MTCs by exome sequencing and found dominant and mutually exclusive oncogenic mutations in *RET* and *RAS* (*H-* and *K-Ras*) genes in 17 sporadic MTCs [54]. As expected, most *Ras *mutations corresponded to mutational hot spots in exons 2 and 3, although some mutations were also detected in exon 4. This study confirms that *RET *and *Ras* mutations are mutually exclusive, and that they are probably 2 different oncogenic driver events in MTC [55]. These results suggest that the activation of the proto-oncogenes *Ras* and *RET *represents alternative genetic events in sporadic MTC tumorigenesis, and that more sensitive sequencing techniques such as next generation sequencing are necessary to detect mutations. For decades, the *Ras* and *Raf* families of oncogenes have been known to be transforming genes. However, in many normal cultured cell types, the sustained expression of activated Ras or its downstream effector, Raf, can elicit cell cycle arrest or senescence. The Ras/Raf-mediated growth arrest has been proposed as a defense mechanism against the inappropriate activation of the Ras/Raf signal transduction pathway [56, 57]. According to this hypothesis, spontaneous mutations in *Ras* genes, which may occur stochastically in all cell types, would be innocuous for the organism because these mutations would lead to growth arrest or senescence. Hence, for carcinogenesis to occur in response to Ras/Raf activation, the growth arrest response must be inactivated. Thus, cell transformation may involve additional events. The growth arrest response to Ras/Raf activation is not limited to normal cells. Several tumor cell lines that were derived from different tumor types, including medullary thyroid carcinoma, also experienced growth arrest, usually accompanied by differentiation or senescence [58–60]. These findings indicate that some tumors retain a capability for growth arrest in response to Ras/Raf activation. The mechanism by which Ras or Raf activation can induce growth arrest is not completely understood. Some investigators have reported that Ras or Raf activation could induce the expression and secretion of a protein that mediated differentiation and G1 cell cycle arrest in MTC cells. By protein purification and mass spectrometry, this protein has been identified as the leukemia inhibitory factor (LIF). STAT3 activation was necessary for LIF-mediated growth arrest and differentiation in MTC cells. In addition, the Ras/Raf/MEK/ERK pathway could also mediate growth arrest and differentiation by a second mechanism that is independent of LIF/JAK/STAT3. This novel autocrine-paracrine mechanism, which mediates crosstalk between the Ras/Raf/MEK/ERK and the JAK-STAT pathways, defines a novel mechanism of Ras/Raf-induced cell growth arrest [61, 62].

Raf-1 activation in human MTC TT cells resulted in the phosphorylation of GSK-3beta. The inactivation of GSK-3beta in TT cells by well-known GSK-3beta inhibitors, such as lithium chloride (LiCl) and SB216763, is associated with both growth suppression and a significant decrease in neuroendocrine markers, such as human achaete-scute complex-like 1 and chromogranin A. Growth inhibition by GSK-3beta inactivation was found to be associated with cell cycle arrest due to an increase in the levels of cyclin-dependent kinase inhibitors, such as p21, p27, and p15. Additionally, TT xenografts mice, treated with LiCl, showed a significant reduction in tumor volume compared with those that were treated with a control. Therefore, GSK-3beta is a key downstream target of the Raf-1 pathway in TT cells, and the inactivation of GSK-3beta alone is sufficient to inhibit the growth of TT cells both *in vitro* and *in vivo *[63]. 

**β*-Catenin Pathway.* *β*-Catenin is a ubiquitously expressed multifunctional protein that plays an important role in cellular adhesion. A novel RET-*β*-Catenin signaling pathway was found to be a critical contributor to enhanced cell proliferation and tumor progression in thyroid cancer. Gujral et al. showed that RET could induce *β*-Catenin-mediated transcription, cell proliferation, and transformation *in vitro* and that *β*-Catenin nuclear localization and subsequent mediation of *β*-Catenin by RET are key secondary events in tumor growth and metastasis *in vivo *[64]. This novel interaction suggests a mechanism that may underlie the broad and early metastatic potential of MTC. These data suggest an unrecognized role for *β*-Catenin signaling that may have implications for tyrosine kinase-mediated tumorigenesis in multiple tumor types and provide another potential target for therapeutic agents. In support, a recent study performed on tissue microarray observed that WNT pathway proteins, including Wisp-1, Wisp-2, and *β*-Catenin, were actively expressed in MTC [17]. 

*NF-*κ*B Pathway.* The nuclear factor kappa-B (NF-*κ*B) proteins, a family of transcription factors that are found virtually in all cells, are known to play crucial roles in the growth of many human malignancies. The ability of NF-*κ*B to target a large number of genes that regulate cell proliferation, differentiation, survival, and apoptosis provides clues towards its dysregulation during the processes of tumorigenesis, metastatic progression, and therapeutic resistance of tumors. NF-*κ*B is constitutively active in MTC through the RET-induced phosphorylation, ubiquitination, and proteosomal degradation of inhibitors of NF-*κ*B (IkB), which allows NF-*κ*B to enter the nucleus and bind to DNA [65]. NF-*κ*B is frequently activated in MTC, and the activation of RET by somatic or germline mutations may be responsible for NF-*κ*B activation in these tumors [5]. These results suggest that the NF-*κ*B pathway may be an important target for drug development in MTC. 

*Novel Protein Targets.* A recent study examined the expression of proteins involved in angiogenesis, inflammation, apoptosis, cell cycle, cell-to-cell contact, and carcinogenesis using high-throughput tissue microarrays from 23 patients with MTC. These authors identified several novel potentially important protein targets such as COX-1/2, Bcl-2a, Gst-*π*, Gli-1, Gli-2, Gli-3, and Bmi-1 that may be therapeutically targeted in MTC. For example, COX-1 and COX-2, which are two inflammation-related factors, were significantly expressed in these cases, suggesting that nonsteroidal anti-inflammatory drugs may provide benefit in some patients with MTC. Then, the finding of antiapoptotic Bcl-2a and Gst-*π* overexpression in MTC suggests that Bcl-2a and Gst-*π* inhibitors might be a treatment option for patients with advanced or metastatic MTC. The same is applied to Gli-1, Gli-2, and Gli-3, members of Sonic Hedgehog Homolog (SHH) pathway, and Bmi-1, a cell-cycle marker that resulted overexpressed in these MTC samples [17]. The studies of these markers, particularly the members of the SHH, may improve our understanding of mechanism of resistance to current chemotherapeutic and/or TKI regimens and identify novel potential therapeutic approaches. 

## 3. Potential Targeted Therapies for MTC

### 3.1. Tyrosine Kinase Receptor Inhibitors

**Figure 1:**
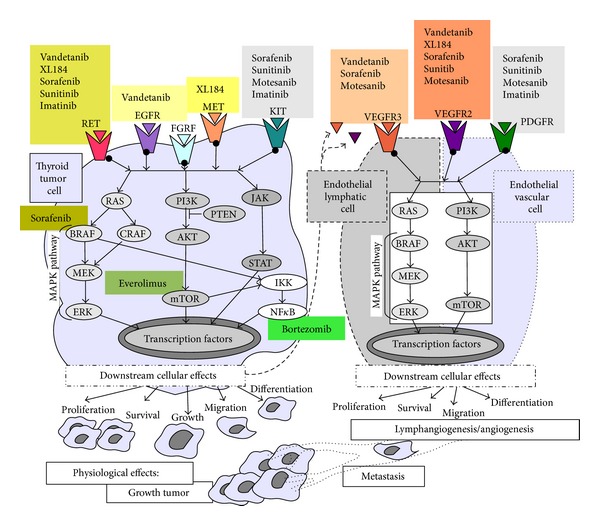
This figure schematically shows the tyrosine kinase receptors, such as RET, EGFR, FGFR, MET, KIT, VEGFR, PDGFR, and some of their downstream effectors. The two most important oncogenic signaling pathways are PI3K/AKT/mTOR and Ras/Raf/MEK/ERK. The activation of the pathways may transduce different transcription factors and induce tumor and endothelial cell proliferation, survival, migration, with subsequent tumor growth, lymphangiogenesis/angiogenesis, and metastasis. Moreover, the main tyrosine kinase inhibitors and their targets are shown.

Due to increased knowledge of the molecular pathogenesis of MTC, therapeutic agents that target specific altered pathways have been developed (Figure 1). Because the alterations of protein kinases and their pathways are involved in MTC development, several tyrosine kinase receptors inhibitors (TKIs) have been tested *in vitro*, preclinical, and clinical studies [66]. RET is certainly an attractive target for several types of tumors particularly for parafollicular C-cells-derived tumors, which are addicted to RET and its activity [66]. TKIs are small organic compounds that affect tyrosine kinase-dependent oncogenic pathways by competing with ATP-binding sites of the tyrosine kinase catalytic domains [67]. Occupation of these sites inhibits autophosphorylation and activation of the tyrosine kinases and prevents the further activation of intracellular signaling pathways. TKIs can be specific to one or several homologous tyrosine kinases. 

Several TKIs that are directed against RET kinase have been developed for the treatment of MTC, but there is no currently available tyrosine kinase inhibitor specific to RET. However, several multitargeted tyrosine kinase inhibitors have demonstrated significant activity against RET (Table 1), including vandetanib, sorafenib, motesanib, imatinib, and sunitinib [66, 68]. The inhibition of only one kinase receptor may induce the compensatory activation of other TKs and consequent resistance to treatment with TKIs [69, 70]. Therefore, the simultaneous inhibition of multiple activated TKs may be the best way to approach MTC [71–73]. TKIs that are currently undergoing testing in clinical trials are described as follows.

*Vandetanib *(ZD6474) is an oral TKI that targets RET, VEGFR-2, and -3, and at higher concentrations, EGFR [74]. Vandetanib selectively inhibits pathways that are critical for tumor growth and angiogenesis without leading to direct cytotoxic effects on tumor or endothelial cells [75]. Most of the mutant RET oncoproteins have demonstrated sensitivity to vandetanib, while mutations in which valine 804 is substituted by either leucine or methionine (as observed in some cases of MEN2A) rendered the RET kinase significantly resistant to vandetanib. This resistance is likely due to steric hindrance, as the Val804Gly mutation increased the sensitivity of RET to vandetanib [76]. When RET activity was inhibited, overstimulation of EGFR was able to partially compensate for RET through a partial rescue of MAPK pathway activation. The inhibition of EGFR by vandetanib has been shown to prevent this rescue of MAPK pathway activation. These data support the idea that the dual inhibition of RET and EGFR is important, as it may overcome the risk of an escape by MTC cells from a blockade of RET through the compensatory overstimulation of EGFR [69]. In addition, the expression of EGFR has been demonstrated in tumor-associated endothelial cells [77]. In this respect, anti-EGFR agents have been shown to block the proliferation and migration of endothelial cells. Therefore, the anti-EGFR activity of vandetanib might result in an additional direct antiangiogenetic mechanism. One phase II clinical trial showed the antitumor activity of vandetanib (300 mg/day) in patients with hereditary MTC. In this study, 20% of the patients showed a partial response to vandetanib (>30% reduction in tumor diameter), while an additional 53% of patients presented with disease stability at 24 weeks. Only 1 patient showed disease progression while receiving this agent. Of interest, this patient was not affected by any of two *RET* mutations correlated to vandetanib resistance in which valine 804 is substituted by leucine or methionine, but by Y791F *RET* germline mutation [78]. Another clinical trial, in which 19 patients with hereditary MTC were treated with vandetanib (100 mg/day), showed similar results [79]. Vandetanib is the only TKI approved for the treatment of symptomatic or progressive MTC in patients with unresectable locally advanced or metastatic disease [80]. The approval of vandetanib in April 2011 by the US Food and Drug Administration (FDA) was based on the results of the phase III clinical trial, “Study D4200C00058,” in which 331 patients with unresectable locally advanced or metastatic MTC were randomly assigned to receive vandetanib (231) or a placebo (100). This study showed that the median progression-free survival duration (PFS) was 11 months longer in the group that received vandetanib than in the placebo control group and that 45% of the patients had an objective response rate (ORR). Common adverse events (any grade) occurred more frequently with vandetanib compared to the placebo and included diarrhea (56% versus 26%), rash (45% versus 11%), nausea (33% versus 16%), hypertension (32% versus 5%), and headache (26% versus 9%) [81]. The impact of overall survival of MTC patients treated with this compound is presently unknown.

*XL184* (cabozantinib) is an oral selective inhibitor of RET, c-MET, and VEGFR-2. c-MET activation triggers tumor growth and angiogenesis. A phase I trial has shown clinical benefits of XL184 in patients with MTC [82]. These results have led to the expansion of an MTC-enriched patient cohort. The phase I trial results indicated that cabozantinib is active in patients with MTC, including those who harbor somatic *RET* mutations and are potentially at high risk for progression and death [83]. A global phase III pivotal study in MTC is ongoing (http://www.ClinicalTrials.gov/ number NCT00704730).

*Sorafenib *(BAY 43-9006)is a multikinase inhibitor that targets BRAF, VEGFR-2, VEGFR-3, KIT (a stem cell growth factor proto-oncogene involved in cell survival and differentiation), and PDGFR. This drug has been shown to strongly inhibit RET kinase activity *in vitro* [84]. A phase II clinical trial, in which sorafenib (400 mg/twice daily) was given to 21 patients with metastatic medullary carcinoma, reported that of the patients with sporadic MTC, 87.5% achieved disease stability and 6.3% demonstrated partial responses. The median PFS was 17.9 months. The treatment was prematurely terminated in MTC hereditary patients due to slow accrual [85]. In a similar trial, all 5 patients treated with sorafenib exhibited partial responses [86]. Recently, a study of sorafenib was conducted on advanced thyroid carcinoma patients, and partial responses were reported in six out of the 12 (50%) patients with MTC, although the small number of patients requires further prospective studies [87]. 

*Sunitinib* (SU011248) is a small molecule inhibitor that targets many of the same protein kinases as sorafenib, including VEGFR, PDGFR, KIT, and RET. In a phase II clinical trial, 33 patients with either well-differentiated thyroid carcinoma or MTC were treated with sunitinib. One patient had a complete response, 28% of the patients had partial responses, and 46% of the patients exhibited disease stability [88]. The intermediate results of the phase II THYSU study also showed the efficacy of sunitinib in advanced medullary thyroid carcinoma. The final results are yet to be released [89].

*Motesanib *(AMG 706) is a multikinase inhibitor that targets VEGFR receptors 1, 2, and 3, PDGFR, and KIT and exerts antiangiogenic and direct antitumor activities [90]. A phase II study performed in 91 patients with locally advanced or metastatic, progressive or symptomatic, MTC demonstrated that although the objective response rate was low, a significant proportion of the MTC patients (81%) achieved disease stability while receiving motesanib [91]. A recent study investigated the effects of motesanib on wild-type and mutant RET activity *in vitro* and on tumor xenograft growth in a mouse model of MTC. The results of this study demonstrated that motesanib inhibited thyroid tumor xenograft growth, predominantly through the inhibition of angiogenesis and possibly via the direct inhibition of VEGFR2 and RET, which were expressed in tumor cells. These data suggest that angiogenic pathways and specifically the VEGF pathway are still important for MTC cells [92]. 

*Imatinib *(STI571) is a TKI that inhibits RET, PDGFR, and KIT. A phase II trial in which imatinib was tested in metastatic MTC patients yielded disappointing results. The patients showed no objective responses; however, a minority of patients achieved disease stability [93].

Several TK inhibitors have been tested in clinical trials, but the most effective inhibitor and whether there is any specificity for particular *RET* mutations remain unknown. A recent study compared the effects of four TKIs (axitinib, sunitinib, vandetanib, and XL184) on cell proliferation, RET expression and autophosphorylation, and ERK activation in cell lines that express MEN2A (MTC-TT) and MEN2B (MZ-CRC-1) mutation. The findings showed that the inhibitors were specific for different mutations, with XL184 being the most potent inhibitor against the MEN2A mutation and vandetanib the most effective against the MEN2B mutation *in vitro*. No TK inhibitor was superior for all tested cell lines, which indicates that mutation-specific therapies could be beneficial in MTC treatment [94].

### 3.2. Other Emerging Therapies for Medullary Thyroid Cancer

#### 3.2.1. Sensitization of Medullary Thyroid Carcinoma to Conventional Cytotoxic Treatments

Conventional chemotherapy has shown limited efficacy against metastatic MTC. One of the mechanisms for the resistance of MTC to chemotherapeutic drugs is multidrug resistance (MDR) [95]. MDR in cancer cells has been attributed to the overexpression of several plasma membrane ATP-dependent efflux pumps, such as MDR-1 [96]. The enzyme cyclooxygenase (COX-2) has been shown to regulate MDR-1 expression in rat mesangial cells [97]. Furthermore, a study has shown that *in vitro* treatment of an MTC-derived cell line with rofecoxib, a COX-2 inhibitor, was able to sensitize MTC cells to doxorubicin [98]. A recent study, performed *in vitro*, has shown that celecoxib, another COX-2 inhibitor, was able to induce both MTC cell apoptosis and sensitization to vinorelbine, thus enhancing the chemotherapeutic effect of this drug [99]. A clinical trial, in which the *in vivo* activities of celecoxib were explored in MTC patients who cannot benefit from available treatments, would be desirable after accounting for the possible cardiovascular risks of this drug.

#### 3.2.2. Drugs That Inhibit MTC Tumorigenesis Targets other than TKRs

Several other therapeutic agents are being investigated for their uses in the treatment of thyroid carcinomas, including MTC. These agents inhibit targets that are involved in development of MTC other than the tyrosine kinase receptors. We previously discussed that RAS operates in a complex signaling network with multiple activators and effectors, which allows them to regulate many cellular functions such as cell proliferation, differentiation, apoptosis, and senescence. Phosphatidylinositol 3-kinase (PI3K) is one of the main effector pathways of RAS, regulating cell growth, cell cycle entry, cell survival, cytoskeleton reorganization, and metabolism. The presence of *Ras* mutations in sporadic MTC and most importantly the frequent activation of the PI3K/AKT/mTOR pathway in several aggressive and metastatic MTCs strongly suggest that this cellular signaling pathway is a good candidate for targeted therapies against MTC. In fact, several *in vitro *evidences have demonstrated that the indirect blocking of this pathway, by PI3K inhibitors [100], or direct inhibition of mTOR [48] can mediate the induction of apoptosis and a decrease in cell viability in the MTC TT cell line. Currently, there are several clinical trials in which patients with radioiodine-refractory differentiated and medullary thyroid carcinomas were recruited to test the efficacy of everolimus (RAD001), a novel inhibitor of mTOR, in combination with other drugs (NCT01625520 and NCT01270321). Everolimus has demonstrated antitumor efficacy in various cancer types, including MTC. Recently, researchers have developed a liposomal form of everolimus and have demonstrated the anticancer efficacy of this formulation against TT cells [101]. Studies have shown that metformin can decrease the proliferation of cancer cells through 5′-AMP-activated protein kinase-(AMPK-) dependent inhibition of mTOR. Some researchers have reported growth inhibitory effects of metformin against MTC cell lines (TT and MZ-CRC-1), which were attributable to the downregulation of both mTOR/6SK and pERK signaling. The expression of the molecular targets of metformin in human MTC cells suggests that this drug may be potentially useful in the treatment of MTC [49]. 

PI3K-AKT-mTOR and RAF-MEK-ERK signaling have been shown to be important in the resistance of thyroid cancer cells to apoptosis and the promotion of tumor progression. In this context, targeted anti-VEGFR therapy or RAF inhibition may be ineffective if PI3K signaling remains intact. Therefore, two promising drugs, RAF265, a RAF inhibitor that is active against VEGFR2, and BEZ235, a PI3K inhibitor, were tested alone and in combination in preclinical MTC models that represented the key genotypes observed in refractory thyroid cancers. The study findings showed that a combination treatment with agents that inhibited both RAF and PI3K pathways strongly inhibited growth both* in vitro *and* in vivo. *In addition, the investigators showed for the first time that RAF265 potently inhibits the constitutively active *RET* ^*C*634*W*^, a form of the kinase that is observed in MEN2A [102].

Persistent RET activation, a frequent event in MTC, leads to the activation of the PI3K/AKT/mTOR, ERK/MAPK, and JAK/STAT3 pathways. Recently, the efficacy of the JAK1/2 inhibitor AZD1480 against the growth of thyroid cancer was tested *in vitro* and* in vivo* in thyroid cancer cell lines that expressed oncogenic RET. The findings showed that AZD1480 efficiently inhibited the growth and tumorigenesis of thyroid cancer cell lines that harbored oncogenic *RET* alterations, likely through the inhibition of PI3K-AKT signaling; this result supports the use of this inhibitor in patients with thyroid cancer, particularly in those with advanced MTC, for whom there are no effective therapeutic options [103].

Several MTCs display a rich but heterogeneous expression of somatostatin receptors (sst1–5) [104]. Thus, somatostatin (SRIF) peptide analogues in combination with TKIs may be a promising approach for the treatment of these tumors. The presence of different combinations of SRIF receptor (SSTR) subtypes in a given patient may explain the variable clinical response to SRIF analogues and may promote the search for more selective drugs with different affinities to the various receptor subtypes [105]. The currently available somatostatin analogues (octreotide and lanreotide) act preferentially through the somatostatin receptor subtype 2 (sst2). In MTC, these compounds have been reported to exert antisecretive effects on calcitonin but unfortunately are not reported to have antiproliferative effects. Pasireotide (SOM230) is a new somatostatin analogue that has a peculiar binding profile with high affinity for sst1, sst2, sst3, and sst5. Preliminary data from a phase II study of patients with metastatic carcinoma show that SOM230 is effective, and some clinical trials are exploring the efficacy of SOM230 alone or in combination with RAD001 in patients with MTC (NCT01625520 and NCT01270321). 

The NF-*κ*B pathway is also a potential target for drug development, and a number of compounds have been developed to inhibit this pathway at different levels in cancer cells. Studies have demonstrated that the proteosome inhibitor bortezomib exerted a promising antitumor effect in human MTC cells through the inhibition of I*κ*B degradation, which led to the inactivation of the transcriptional factor NF-*κ*B [106]. Patients are currently being recruited for a phase I/II trial to study the combination of vandetanib plus bortezomib (http://www.ClinicalTrials.gov/). Patients with MTC will participate in the phase II study [21].

#### 3.2.3. Immunotherapy and Radioimmunotherapy

Immunization with tumor antigen-pulsed autologous dendritic cells (DCs) resulted in protective immunity and the rejection of various established human tumors. Specifically, vaccination immunotherapy with calcitonin and/or carcinoembryonic antigen (CEA) peptide-pulsed DCs was shown to result in the induction of a cellular, antigen-specific immune response in patients with MTC, which led to clinical responses in some patients. Therefore, for the first time, a potential DC vaccination therapy was developed for patients with metastatic MTC [107]. Another study, which was performed in a transgenic MTC mouse model, has confirmed this finding [108]. 

Papewalis et al. have reported on the *in vitro* findings of a vaccination trial in 5 MTC patients, who were treated with DCs that were generated using a new protocol, which consisted of granulocyte-macrophage colony-stimulating factor and interferon-alpha (IFN-DCs). These investigators demonstrated that immunization with IFN-DCs led to a tumor epitope-specific Th1 immune response in MTC patients [109]. Furthermore, a pilot trial of 10 patients has assessed the safety and efficacy, in terms of immune responses and clinical activities, of the DCs. In this study, DCs were injected into groin lymph nodes at 3-week intervals. Monitoring of the patients included serial measurements of calcitonin tumor markers, radiological imaging, and immunological *in vitro* tests, including T-cell interferon-gamma detection and cytotoxicity assays. DC vaccinations were determined to be well tolerated and safe. After a median followup of 11 months (range 7–26), 3 (30%) of the 10 patients exhibited disease stability, while 7 (70%) of the patients progressed during treatment. A combined treatment with different tumor cell lysate-pulsed DCs increased the likelihood of a calcitonin tumor marker response and should therefore be preferred over monotherapy with DCs pulsed with a single lysate [110]. 

Thus, immunotherapy may be a promising alternative therapy in combination with agents that target specific signal transduction pathways for aggressive forms of MTC that are resistant to classical therapies.

A significant antitumor effect was also observed with radioimmunotherapy using an anti-CEA ^131^I-F6 monoclonal antibody in MTC-bearing nude mice. Nevertheless, no complete responses were observed. Similarly to chemotherapy, drugs that target the tumor microenvironment might improve the efficacy of radioimmunotherapy. This hypothesis was confirmed by a recent study in which pretreatment of mice grafted with the TT human medullary thyroid cancer cell line with antiangiogenic therapies was found to improve the efficacy of radioimmunotherapy with acceptable toxicity [111]. Another independent study, which was conducted in a mouse model of MTC, has found similar results with the angiogenesis inhibitor bevacizumab [112]. Future investigations will be performed to better understand how antiangiogenic agents enhance the efficacy of radioimmunotherapy.

#### 3.2.4. Epigenetic Therapy

Epigenetic drugs are expected to target the two main mechanisms of epigenetic alterations, DNA methylation and acetylation, and are regarded with increasing interest by both endocrinologists and oncologists. Concluded trials of such drugs have shown that few patients with advanced thyroid cancer responded completely, which suggests that these treatments were effective at stabilizing progressive disease. Therefore, definitive results from clinical trials will clarify the true effectiveness of epigenetic drugs in these tumors. Epigenetic drugs, when used in combination with other target molecules, might significantly increase response rates to treatment in advanced thyroid cancer patients, either by relaxing the chromatin structure to make DNA more accessible to the effects of a DNA targeting drug or by acting synergistically with antimitotic drugs [113]. Thus, epigenetic therapy may be a promising novel approach for the treatment of some cases of MTC. 

### 3.3. Potential Mechanisms of Resistance to Therapy in MTC

Tumor cells often devise strategies to bypass the effects of antineoplastic agents, and the selection of therapy-resistant clones is frequently the reason for treatment failure. One characteristic of endocrine cancer is a general resistance to conventional chemotherapies or radiotherapies that would normally lead to apoptosis of the cancer cells. MTC can develop resistances to cytotoxic drugs due to the expression of the multidrug resistance (MDR) 1 gene. Drugs that can oppose this mechanism of resistance to traditional chemotherapies may increase the sensitivity of MTC tumor cells to chemotherapy. For example, celecoxib, a COX-2 inhibitor, has been shown to potentiate the chemotherapeutic effect of vinorelbine in MTC [99]. Therefore, the synergistic actions of a cytotoxic drug and a compound that increases the sensitivity of MTC cells to such a drug could provide a treatment method for patients who cannot benefit from TKIs. However, the most promising results in patients with chemotherapy and radiotherapy-unresponsive MTC were obtained with TKIs. The inhibition of a single RTK may engage compensatory signaling that maintains cell growth. Multitargeted tyrosine kinase inhibitors, which inhibit multiple RTKs simultaneously, including RET, have been developed to bypass this potential resistance mechanism. One complication is that such inhibitors may not only be more effective for targeting receptors in tumor cells but may also exhibit greater toxicity. Thus, the challenge is to balance the increased efficacy of these inhibitors with the potential for a broader array of side effects [7]. Moreover, some MTC patients cannot take advantage of these therapies due to specific *RET* mutations that confer resistance to TKIs (e.g., RET V804 confers resistance to vandetanib) [76, 114]. Finally an important study reported the existence of cancer stem-like cells in MTC, which exhibit the features of self-renewal and of multiple lineage differentiation that is dependent on RET proto-oncogene receptor activity suggesting a potential mechanism of resistance to cytotoxic or TKIs agents [115].

## 4. Conclusions

Most cases of metastatic MTC are incurable due to resistance to conventional chemo- and radiotherapies. In recent decades, the identification of genetic defects and altered cellular signaling pathways involved in human MTC tumorigenesis have led to the development of targeted therapies, of which the most important are tyrosine kinase inhibitors. However, the low rates of partial responses or complete responses and the short duration of responses in MTC patients who were observed while taking these drugs prompted researchers to develop new drugs and alternative therapies to be combined with multi-TKIs and to reduce potential cross-toxicity effects. To this end, a recent study has shown synergistic effects with a combination of sorafenib and the MEK inhibitor AZD6244 against a human MTC cell line. Concomitant use of a RAF inhibitor, RAF265, and a dual PI3K/mTOR inhibitor, BEZ-235, was another effective combinatorial therapy against thyroid cancer in xenograft mouse models [102]. The mTOR cascade is emerging probably as one of the most important deregulated pathways in advanced and metastatic MTC and certainly deserves further study. Targeting mTOR in combination might be efficacious in patients with this tumor types.

However, a complete overview of all signaling pathways, including their interactions, role of the activating gene mutations that contribute to MTC tumorigenesis, and mechanisms of intrinsic and acquired resistance to treatments, is required and will permit us to identify the best therapy for each patient. Novel combined or sequential therapies request a further step in the knowledge of the cellular signaling of this tumor. Finally, larger multi-institutional clinical trials to fully understand the clinical benefits of these therapies are warranted.

## Figures and Tables

**Table 1:** Main targeted therapies currently in use for the treatment of medullary thyroid cancer.

		Key targets										http://www.clinicaltrials.gov/	
	Drug name										IC_50_ RET [*μ*M]*
		RET	EGFR	VEGFRs	PDGFR	cKIT	FGFR	MET	BRAF	Other		Drugs in combination	Status identifier (phase)
	Vandetanib (ZD6474)	*√*	*√*	*√*	*√*						0.1	Bortezomib	*Not yet recruiting*: NCT01661179 (1,2) *Recruiting*: NCT00514046 (1,2) NCT01496313 (4) *Active, not recruiting, has * *results*: NCT00410761 (3) NCT00358956 (2) NCT00098345 (2) *Active, not recruiting*: NCT000923247 (1,2) NCT01298323 (3) *Withdrawn*:
												Docetaxel	NCT000937417
	XL184 (Cabozantinib)	*√*		*√*		*√*		*√*		*√*	0.004		*Active, not recruiting*: NCT00704730 (3) NCT00215605 (1) *Available*: NCT01683110
Main TKIs	Sorafenib (BAY-43-9006)	*√*		*√*	*√*	*√*	*√*		*√*	*√*	0.0059–0.05		*Active, not recruiting*: NCT00390325 (2) Unknown **: NCT006542387 (2)
	Sunitinib (SU11248)	*√*		*√*	*√*	*√*				*√*	0.22–1.3		*Active, not recruiting*: NCT00381641 (2) NCT00519896 (2) NCT00510640 (2)
	Motesanib (AMG-706)	*√*		*√*	*√*	*√*					0.05–0.09		*Completed*: NCT00121628 (2)
	Imatinib (STI571)				*√*	*√*				*√*	5–37	Xeloda, Dacarbazine	*Active, not recruiting*: NCT00354523 (1,2)

	Everolimus (RAD001)	mTOR										Pasireotide Pasireotide	*Recruiting*: NCT01625520 (2) NCT01270321 (2) NCT01164176 (2) Unknown **: NCT0111865 (2)
Main Non-TKIs	Bortezomib (PS-341)	proteosome										Vandetanib	*Active, not recruiting*: NCT00923247 (1,2)
	Pasireotide (SOM230)	sst1, 3, 5 (somatostatin receptors)										Everolimus Everolimus	*Recruiting*: NCT01625520 (2) NCT01270321 (2)

*IC_50_ RET [*μ*M]: 50% inhibitory concentration.

**Status has not been verified in more than two years.

## References

[1] Antonelli A, Martina Ferrari S, Fallahi P (2010). Medullary thyroid cancer: new targeted molecular therapies. *Recent Patents on Endocrine, Metabolic and Immune Drug Discovery*.

[2] Ball DW, Baylin SB, De Butros AC, Braverman LE, Utiger RD (2000). Medullary thyroid carcinoma. *Werner and Ingbar’s the Thyroid*.

[3] Pinchera A, Ernest lM, Ujjal CH, Mallick K, Kendall-Taylor P (2006). Medullary thyroid cancer: diagnosis and treatment. *Practical Management of Thyroid Cancer*.

[4] Roman S, Lin R, Sosa JA (2006). Prognosis of medullary thyroid carcinoma: demographic, clinical, and pathologic predictors of survival in 1252 cases. *Cancer*.

[5] Santarpia L, Ye L, Gagel RF (2009). Beyond RET: potential therapeutic approaches for advanced and metastatic medullary thyroid carcinoma. *Journal of Internal Medicine*.

[6] Brassard M, Rondeau G (2012). Role of vandetanib in the management of medullary thyroid cancer. *Biologics*.

[7] Ye L, Santarpia L, Gagel RF (2010). The evolving field of tyrosine kinase inhibitors in the treatment of endocrine tumors. *Endocrine Reviews*.

[8] Husain M, Alsever RN, Lock JP (1978). Failure of medullary carcinoma of the thyroid to respond to doxorubicin therapy. *Hormone Research*.

[9] Droz JP, Schlumberger M, Rougier P, Ghosn M, Gardet P, Parmentier C (1990). Chemotherapy in metastatic nonanaplastic thyroid cancer: experience at the Institut Gustave-Roussy. *Tumori*.

[10] Scherübl H, Raue F, Ziegler R (1990). Combination chemotherapy of advanced medullary and differentiated thyroid cancer. Phase II study. *Journal of Cancer Research and Clinical Oncology*.

[11] Antonelli A, Fallahi P, Ferrari SM (2012). RET TKI: potential role in thyroid cancers. *Current Oncology Reports*.

[12] Lodish MB, Stratakis CA (2008). RET oncogene in MEN2, MEN2B, MTC and other forms of thyroid cancer. *Expert Review of Anticancer Therapy*.

[13] Airaksinen MS, Saarma M (2002). The GDNF family: signalling, biological functions and therapeutic value. *Nature Reviews Neuroscience*.

[14] Drosten M, Pützer BM (2006). Mechanisms of disease: cancer targeting and the impact of oncogenic RET for medullary thyroid carcinoma therapy. *Nature Clinical Practice Oncology*.

[15] Liu Z, Hou P, Ji M (2008). Highly prevalent genetic alterations in receptor tyrosine kinases and phosphatidylinositol 3-kinase/Akt and mitogen-activated protein kinase pathways in anaplastic and follicular thyroid cancers. *Journal of Clinical Endocrinology and Metabolism*.

[16] Mitsiades CS, Kotoula V, Poulaki V (2006). Epidermal growth factor receptor as a therapeutic target in human thyroid carcinoma: mutational and functional analysis. *Journal of Clinical Endocrinology and Metabolism*.

[17] Erovic BM, Kim D, Cassol C (2012). Prognostic and predictive markers in medullary thyroid carcinoma. *Endocrine Pathology*.

[18] Rodríguez-Antona C, Pallares J, Montero-Conde C (2010). Overexpression and activation of EGFR and VEGFR2 in medullary thyroid carcinomas is related to metastasis. *Endocrine-Related Cancer*.

[19] Croyle M, Akeno N, Knauf JA (2008). RET/PTC-induced cell growth is mediated in part by epidermal growth factor receptor (EGFR) activation: evidence for molecular and functional interactions between RET and EGFR. *Cancer Research*.

[20] Capp C, Wajner SM, Siqueira DR, Brasil BA, Meurer L, Maia AL (2010). Increased expression of vascular endothelial growth factor and its receptors, VEGFR-1 and VEGFR-2, in medullary thyroid carcinoma. *Thyroid*.

[21] Kerbel RS (2008). Tumor angiogenesis. *New England Journal of Medicine*.

[22] Gómez K, Varghese J, Jiménez C (2011). Medullary thyroid carcinoma: molecular signaling pathways and emerging therapies. *Journal of Thyroid Research*.

[23] Jeffers M, Fiscella M, Webb CP, Anver M, Koochekpour S, Vande Woude GF (1998). The mutationally activated Met receptor mediates motility and metastasis. *Proceedings of the National Academy of Sciences of the United States of America*.

[24] Papotti M, Olivero M, Volante M (2000). Expression of hepatocyte growth factor (HGF) and its receptor (MET) in medullary carcinoma of the thyroid. *Endocrine Pathology*.

[25] Wasenius VM, Hemmer S, Karjalainen-Lindsberg ML, Nupponen NN, Franssila K, Joensuu H (2005). MET receptor tyrosine kinase sequence alterations in differentiated thyroid carcinoma. *American Journal of Surgical Pathology*.

[26] Ivan M, Bond JA, Prat M, Comoglio PM, Wynford-Thomas D (1997). Activated ras and ret oncogenes induce over-expression of c-met (hepatocyte growth factor receptor) in human thyroid epithelial cells. *Oncogene*.

[27] Bernard RS, Zheng L, Liu W, Winer D, Asa SL, Ezzat S (2005). Fibroblast growth factor receptors as molecular targets in thyroid carcinoma. *Endocrinology*.

[28] Ezzat S, Huang P, Dackiw A, Asa SL (2005). Dual inhibition of RET and FGFR4 restrains medullary thyroid cancer cell growth. *Clinical Cancer Research*.

[29] Matsuo K, Tang SH, Sharifi B, Rubin SA, Schreck R, Fagin JA (1993). Growth factor production by human thyroid carcinoma cells: abundant expression of a platelet-derived growth factor-B-like protein by a human papillary carcinoma cell line. *Journal of Clinical Endocrinology and Metabolism*.

[30] Cerrato A, De Falco V, Santoro M (2009). Molecular genetics of medullary thyroid carcinoma: the quest for novel therapeutic targets. *Journal of Molecular Endocrinology*.

[31] Harrison DJ, Hooper ML, Armstrong JF, Clarke AR (1995). Effects of heterozygosity for the Rb-1(t19neo) allele in the mouse. *Oncogene*.

[32] Williams BO, Remington L, Albert DM, Mukai S, Bronson RT, Jacks T (1994). Cooperative tumorigenic effects of germline mutations in Rb and p53. *Nature Genetics*.

[33] Harvey M, Vogel H, Lee EYHP, Bradley A, Donehower LA (1995). Mice deficient in both p53 and Rb develop tumors primarily of endocrine origin. *Cancer Research*.

[34] Coxon AB, Ward JM, Geradts J, Otterson GA, Zajac-Kaye M, Kaye FJ (1998). RET cooperates with RB/p53 inactivation in a somatic multi-step model for murine thyroid cancer. *Oncogene*.

[35] Joshi PP, Kulkarni MV, Yu BK (2007). Simultaneous downregulation of CDK inhibitors p18Ink4c and p27Kip1 is required for MEN2A-RET-mediated mitogenesis. *Oncogene*.

[36] Segouffin-Cariou C, Billaud M (2000). Transforming ability of MEN2A-RET requires activation of the phosphatidylinositol 3-kinase/AKT signaling pathway. *Journal of Biological Chemistry*.

[37] Pitt SC, Chen H (2008). The phosphatidylinositol 3-kinase/akt signaling pathway in medullary thyroid cancer. *Surgery*.

[38] Kouvaraki MA, Liakou C, Paraschi A (2011). Activation of mTOR signaling in medullary and aggressive papillary thyroid carcinomas. *Surgery*.

[39] Asai N, Murakami H, Iwashita T, Takahashi M (1996). A mutation at tyrosine 1062 in MEN2A-Ret and MEN2B-Ret impairs their transforming activity and association with Shc adaptor proteins. *Journal of Biological Chemistry*.

[40] Murakami H, Iwashita T, Asai N (1999). Enhanced phosphatidylinositol 3-kinase activity and high phosphorylation state of its downstream signalling molecules mediated by Ret with the MEN 2B mutation. *Biochemical and Biophysical Research Communications*.

[41] Salvatore D, Melillo RM, Monaco C (2001). Increased in vivo phosphorylation of ret tyrosine 1062 is a potential pathogenetic mechanism of multiple endocrine neoplasia type 2B. *Cancer Research*.

[42] Pelicci G, Troglio F, Bodini A (2002). The neuron-specific Rai (ShcC) adaptor protein inhibits apoptosis by coupling ret to the phosphatidylinositol 3-kinase/Akt signaling pathway. *Molecular and Cellular Biology*.

[43] Sansal I, Sellers WR (2004). The biology and clinical relevance of the PTEN tumor suppressor pathway. *Journal of Clinical Oncology*.

[44] Zbuk KM, Eng C (2007). Cancer phenomics: RET and PTEN as illustrative models. *Nature Reviews Cancer*.

[45] Yuan TL, Cantley LC (2008). PI3K pathway alterations in cancer: variations on a theme. *Oncogene*.

[46] Rapa I, Saggiorato E, Giachino D (2011). Mammalian target of rapamycin pathway activation is associated to RET mutation status in medullary thyroid carcinoma. *Journal of Clinical Endocrinology and Metabolism*.

[47] Tamburrino A, Molinolo AA, Salerno P (2012). Activation of the mTOR pathway in primary medullary thyroid carcinoma and lymph node metastases. *Clinical Cancer Research*.

[48] Grozinsky-Glasberg S, Rubinfeld H, Nordenberg Y (2010). The rapamycin-derivative RAD001 (everolimus) inhibits cell viability and interacts with the Akt-mTOR-p70S6K pathway in human medullary thyroid carcinoma cells. *Molecular and Cellular Endocrinology*.

[49] Klubo-Gwiezdzinska J, Jensen K, Costello J (2012). Metformin inhibits growth and decreases resistance to anoikis in medullary thyroid cancer cells. *Endocrine Related Cancer*.

[50] Bockhorn M, Frilling A, Kalinin V, Schroder S, Broelsch CE (2000). Absence of H- and K-ras oncogene mutations in sporadic medullary thyroid carcinoma. *Experimental and Clinical Endocrinology and Diabetes*.

[51] Schulten HJ, Al-Maghrabi J, Al-Ghamdi K (2011). Mutational screening of RET, HRAS, KRAS, NRAS, BRAF, AKT1, and CTNNB1 in medullary thyroid carcinoma. *Anticancer Research*.

[52] Ciampi R, Mian C, Fugazzola L (2013). Evidence of a low prevalence of Ras mutations in a large medullary thyroid cancer series. *Thyroid*.

[53] Moura MM, Cavaco BM, Pinto AE, Leite V (2011). High prevalence of RAS mutations in RET-negative sporadic medullary thyroid carcinomas. *Journal of Clinical Endocrinology and Metabolism*.

[54] Agrawal N, Jiao Y, Sausen M (2012). Exomic sequencing of medullary thyroid cancer reveals dominant and mutually exclusive oncogenic mutations in RET and RAS. *Journal of Clinical Endocrinology and Metabolism*.

[55] Boichard A, Croux L, Al Ghuzlan A (2012). Somatic Ras mutations occur in a large proportion of sporadic RET-negative medullary thyroid carcinomas and extend to a previously unidentified exon. *The Journal of Clinical Endocrinology and Metabolism*.

[56] Weinberg RA (1997). The cat and mouse games that genes, viruses, and cells play. *Cell*.

[57] Santarpia L, Lippman SM, El_Naggar AK (2012). Targeting the MAPK-RAS-RAF signaling pathway in cancer therapy. *Expert Opinion on Therapeutic Targets*.

[58] Carson EB, McMahon M, Baylin SB, Nelkin BD (1995). Ret gene silencing is associated with raf-1-induced medullary thyroid carcinoma cell differentiation. *Cancer Research*.

[59] Shirasawa S, Furuse M, Yokoyama N, Sasazuki T (1993). Altered growth of human colon cancer cell lines disrupted at activated Ki-ras. *Science*.

[60] Wood KW, Qi H, D’Arcangelo G, Armstrong RC, Roberts TM, Halegoua S (1993). The cytoplasmic raf oncogene induces a neuronal phenotype in PC12 cells: a potential role for cellular raf kinases in neuronal growth factor signal transduction. *Proceedings of the National Academy of Sciences of the United States of America*.

[61] Park JI, Strock CJ, Ball DW, Nelkin BD (2003). The Ras/Raf/MEK/extracellular signal-regulated kinase pathway induces autocrine-paracrine growth inhibition via the leukemia inhibitory factor/JAK/STAT pathway. *Molecular and Cellular Biology*.

[62] Arthan D, Hong SK, Park JI (2010). Leukemia inhibitory factor can mediate Ras/Raf/MEK/ERK-induced growth inhibitory signaling in medullary thyroid cancer cells. *Cancer Letters*.

[63] Kunnimalaiyaan M, Vaccaro AM, Ndiaye MA, Chen H (2007). Inactivation of glycogen synthase kinase-3*β*, a downstream target of the raf-1 pathway, is associated with growth suppression in medullary thyroid cancer cells. *Molecular Cancer Therapeutics*.

[64] Gujral TS, Van Veelen W, Richardson DS (2008). A novel RET kinase-*β*-catenin signaling pathway contributes to tumorigenesis in thyroid carcinoma. *Cancer Research*.

[65] Ludwig L, Kessler H, Wagner M (2001). Nuclear factor-*κ*B is constitutively active in C-cell carcinoma and required for RET-induced transformation. *Cancer Research*.

[66] Santarpia L, Bottai G (2012). Inhibition of RET activated pathways: novel strategies for therapeutic intervention in human cancers. *Current Pharmaceutical Design*.

[67] Lorusso PM, Eder JP (2008). Therapeutic potential of novel selective-spectrum kinase inhibitors in oncology. *Expert Opinion on Investigational Drugs*.

[68] Walsh S, Prichard R, Hill AD (2012). Emerging therapies for thyroid carcinoma. *Surgeon*.

[69] Vitagliano D, De Falco V, Tamburrino A (2011). The tyrosine kinase inhibitor ZD6474 blocks proliferation of RET mutant medullary thyroid carcinoma cells. *Endocrine-Related Cancer*.

[70] Stommel JM, Kimmelman AC, Ying H (2007). Coactivation of receptor tyrosine kinases affects the response of tumor cells to targeted therapies. *Science*.

[71] Ocana A, Amir E, Seruga B, Pandiella A (2010). Do we have to change the way targeted drugs are developed?. *Journal of Clinical Oncology*.

[72] van Amerongen R, Berns A (2008). Targeted anticancer therapies: mouse models help uncover the mechanisms of tumor escape. *Cancer Cell*.

[73] Knight ZA, Lin H, Shokat KM (2010). Targeting the cancer kinome through polypharmacology. *Nature Reviews Cancer*.

[74] Sherman SI (2011). Targeted therapies for thyroid tumors. *Modern Pathology*.

[75] Herbst RS, Heymach JV, O’Reilly MS, Onn A, Ryan AJ (2007). Vandetanib (ZD6474): an orally available receptor tyrosine kinase inhibitor that selectively targets pathways critical for tumor growth and angiogenesis. *Expert Opinion on Investigational Drugs*.

[76] Carlomagno F, Guida T, Anaganti S (2004). Disease associated mutations at valine 804 in the RET receptor tyrosine kinase confer resistance to selective kinase inhibitors. *Oncogene*.

[77] De Luca A, Carotenuto A, Rachiglio A (2008). The role of the EGFR signaling in tumor microenvironment. *Journal of Cellular Physiology*.

[78] Wells SA, Gosnell JE, Gagel RF (2010). Vandetanib for the treatment of patients with locally advanced or metastatic hereditary medullary thyroid cancer. *Journal of Clinical Oncology*.

[79] Robinson BG, Paz-Ares L, Krebs A, Vasselli J, Haddad R (2010). Vandetanib (100 mg) in patients with locally advanced or metastatic hereditary medullary thyroid cancer. *Journal of Clinical Endocrinology and Metabolism*.

[80] Commander H, Whiteside G, Perry C (2011). Vandetanib: first global approval. *Drugs*.

[81] Wells SA, Robinson BG, Gagel RF (2012). Vandetanib in patients with locally advanced or metastatic medullary thyroid cancer: a randomized, double-blind phase III trial. *Journal of Clinical Oncology*.

[82] Kurzrock R, Cohen EE, Sherman SI (2010). Long-term results in a cohort of medullary thyroid cancer (MTC) patients (pts) in a phase I study with XL-184 (BMS, 907351), an oral inhibitor of MET, VEGFR2, and RET. *Journal of Clinical Oncology*.

[83] Kurzrock R, Sherman SI, Ball DW (2011). Activity of XL184 (cabozantinib), an oral tyrosine kinase inhibitor, in patients with medullary thyroid cancer. *Journal of Clinical Oncology*.

[84] Carlomagno F, Anaganti S, Guida T (2006). BAY 43-9006 inhibition of oncogenic RET mutants. *Journal of the National Cancer Institute*.

[85] Lam ET, Ringel MD, Kloos RT (2010). Phase II clinical trial of sorafenib in metastatic medullary thyroid cancer. *Journal of Clinical Oncology*.

[86] Frank-Raue K, Ganten M, Kreissl MC, Raue F (2011). Rapid response to sorafenib in metastatic medullary thyroid carcinoma. *Experimental and Clinical Endocrinology and Diabetes*.

[87] Capdevila J, Iglesias L, Halperin I (2010). Sorafenib in patients (pts) with advanced thryroid carcinoma (TC): a compassionate use program. *Journal of Clinical Oncology*.

[88] Carr LL, Mankoff DA, Goulart BH (2010). Phase II study of daily sunitinib in FDG-PET-positive, iodine-refractory differentiated thyroid cancer and metastatic medullary carcinoma of the thyroid with functional imaging correlation. *Clinical Cancer Research*.

[89] Ravaud A, De La Fouchardière C, Asselineau J (2010). Efficacy of sunitinib in advanced medullary thyroid carcinoma: intermediate results of phase II THYSU. *Oncologist*.

[90] Polverino A, Coxon A, Starnes C (2006). AMG 706, an oral, multikinase inhibitor that selectively targets vascular endothelial growth factor, platelet-derived growth factor, and kit receptors, potently inhibits angiogenesis and induces regression in tumor xenografts. *Cancer Research*.

[91] Schlumberger MJ, Elisei R, Bastholt L (2009). Phase II study of safety and efficacy of motesanib in patients with progressive or symptomatic, advanced or metastatic medullary thyroid cancer. *Journal of Clinical Oncology*.

[92] Coxon A, Bready J, Kaufman S (2012). Anti-tumor activity of motesanib in a medullary thyroid cancer model. *Journal of Endocrinological Investigation*.

[93] de Groot JWB, Zonnenberg BA, Van Ufford-Mannesse PQ (2007). A phase II trial of imatinib therapy for metastatic medullary thyroid carcinoma. *Journal of Clinical Endocrinology and Metabolism*.

[94] Verbeek HHG, Alves MM, De Groot JWB (2011). The effects of four different tyrosine kinase inhibitors on medullary and papillary thyroid cancer cells. *Journal of Clinical Endocrinology and Metabolism*.

[95] Massart C, Gibassier J, Lucas C, Pourquier P, Robert J (1996). Doxorubicine resistance modulation by ciclosporin a and verapamil in five human cell lines or medullary thyroid cancer. *Bulletin du Cancer*.

[96] Breier A, Barančík M, Sulová Z, Uhrík B (2005). P-glycoprotein-Implications of metabolism of neoplastic cells and cancer therapy. *Current Cancer Drug Targets*.

[97] Patel VA, Dunn MJ, Sorokin A (2002). Regulation of MDR-1 (P-glycoprotein) by cyclooxygenase-2. *Journal of Biological Chemistry*.

[98] Zatelli MC, Luchin A, Piccin D (2005). Cyclooxygenase-2 inhibitors reverse chemoresistance phenotype in medullary thyroid carcinoma by a permeability glycoprotein-mediated mechanism. *Journal of Clinical Endocrinology and Metabolism*.

[99] Vivaldi A, Ciampi R, Tacito A (2012). Colecoxib, a cyclooxygenase-2 inhibitor, potentiates the chemotherapic effect of vinorelbine in the medullary thyroid cancer TT cell line. *Molecular and Cellular Endocrinology*.

[100] Kunnimalaiyaan M, Ndiaye M, Chen H (2006). Apoptosis-mediated medullary thyroid cancer growth suppression by the PI3K inhibitor LY294002. *Surgery*.

[101] Iwase Y, Maitani Y (2012). Preparation and in vivo evaluation of liposomal everolimus for lung carcinoma and thyroid carcinoma. *Biological & Pharmaucetical Bulletin*.

[102] Jin N, Jiang T, Rosen DM, Nelkin BD, Ball DW (2011). Synergistic action of a RAF inhibitor and a dual PI3K/mTOR inhibitor in thyroid cancer. *Clinical Cancer Research*.

[103] Couto JP, Almeida A, Daly L, Sobrinho-Simões M, Bromberg JF, Soares P (2012). AZD1480 blocks growth and tumorigenesis of RET-activated thryoid cancer cell lines. *PloS One*.

[104] Papotti M, Kumar U, Volante M, Pecchioni C, Patel YC (2001). Immunohistochemical detection of somatostatin receptor types 1–5 in medullary carcinoma of the thyroid. *Clinical Endocrinology*.

[105] Mato E, Matías-Guiu X, Chico A (1998). Somatostatin and somatostatin receptor subtype gene expression in medullary thyroid carcinoma. *Journal of Clinical Endocrinology and Metabolism*.

[106] Mitsiades CS, McMillin D, Kotoula V (2006). Antitumor effects of the proteasome inhibitor bortezomib in medullary and anaplastic thyroid carcinoma cells in vitro. *Journal of Clinical Endocrinology and Metabolism*.

[107] Schott M, Seissler J, Lettmann M, Fouxon V, Scherbaum WA, Feldkamp J (2001). Immunotherapy for medullary thyroid carcinoma by dendritic cell vaccination. *Journal of Clinical Endocrinology and Metabolism*.

[108] Wuttke M, Papewalis C, Meyer Y (2008). Amino acid-modified calcitonin immunization induces tumor epitope-specific immunity in a transgenic mouse model for medullary thyroid carcinoma. *Endocrinology*.

[109] Papewalis C, Wuttke M, Jacobs B (2008). Dendritic cell vaccination induces tumor epitope-specific Th1 immune response in medullary thyroid carcinoma. *Hormone and Metabolic Research*.

[110] Bachleitner-Hofmann T, Friedl J, Hassler M (2009). Pilot trial of autologous dendritic cells loaded with tumor lysate(s) from allogeneic tumor cell lines in patients with metastatic medullary thyroid carcinoma. *Oncology Reports*.

[111] Kraeber-Bodéré F, Bodet-Milin C, Niaudet C (2010). Comparative toxicity and efficacy of combined radioimmunotherapy and antiangiogenic therapy in carcinoembryonic antigen-expressing medullary thyroid cancer xenograft. *Journal of Nuclear Medicine*.

[112] Salaun PY, Bodet-Milin C, Frampas E (2010). Toxicity and efficacy of combined radioimmunotherapy and bevacizumab in a mouse model of medullary thyroid carcinoma. *Cancer*.

[113] Catalano MG, Fortunati N, Boccuzzi G (2012). Epigenetics modifications and therapeutic prospects in human thyroid cancer. *Frontiers in Endocrinology*.

[114] Romei C, Mariotti S, Fugazzola L (2010). Multiple endocrine neoplasia type 2 syndromes (MEN 2): results from the ItaMEN network analysis on the prevalence of different genotypes and phenotypes. *European Journal of Endocrinology*.

[115] Zhu W, Hai T, Ye L, Cote GJ (2010). Medullary thyroid carcinoma cell lines contain a self-renewing CD133 + population that is dependent on Ret proto-oncogene activity. *Journal of Clinical Endocrinology and Metabolism*.

